# Induction of Antibody Responses to African Horse Sickness Virus (AHSV) in Ponies after Vaccination with Recombinant Modified Vaccinia Ankara (MVA)

**DOI:** 10.1371/journal.pone.0005997

**Published:** 2009-06-22

**Authors:** Rachael Chiam, Emma Sharp, Sushila Maan, Shujing Rao, Peter Mertens, Barbara Blacklaws, Nick Davis-Poynter, James Wood, Javier Castillo-Olivares

**Affiliations:** 1 Animal Health Trust, Lanwades Park, Kentford, Newmarket, Suffolk, United Kingdom; 2 Institute for Animal Health, Pirbright Laboratory, Pirbright, Surrey, United Kingdom; 3 Cambridge Infectious Diseases Consortium, Department of Veterinary Medicine, Cambridge, United Kingdom; 4 Sir Albert Sakzewski Virus Research Centre, University of Queensland, Herston, Queensland, Australia; New York University School of Medicine, United States of America

## Abstract

**Background:**

African horse sickness virus (AHSV) causes a non-contagious, infectious disease in equids, with mortality rates that can exceed 90% in susceptible horse populations. AHSV vaccines play a crucial role in the control of the disease; however, there are concerns over the use of polyvalent live attenuated vaccines particularly in areas where AHSV is not endemic. Therefore, it is important to consider alternative approaches for AHSV vaccine development. We have carried out a pilot study to investigate the ability of recombinant modified vaccinia Ankara (MVA) vaccines expressing VP2, VP7 or NS3 genes of AHSV to stimulate immune responses against AHSV antigens in the horse.

**Methodology/Principal Findings:**

VP2, VP7 and NS3 genes from AHSV-4/Madrid87 were cloned into the vaccinia transfer vector pSC11 and recombinant MVA viruses generated. Antigen expression or transcription of the AHSV genes from cells infected with the recombinant viruses was confirmed. Pairs of ponies were vaccinated with MVAVP2, MVAVP7 or MVANS3 and both MVA vector and AHSV antigen-specific antibody responses were analysed. Vaccination with MVAVP2 induced a strong AHSV neutralising antibody response (VN titre up to a value of 2). MVAVP7 also induced AHSV antigen–specific responses, detected by western blotting. NS3 specific antibody responses were not detected.

**Conclusions:**

This pilot study demonstrates the immunogenicity of recombinant MVA vectored AHSV vaccines, in particular MVAVP2, and indicates that further work to investigate whether these vaccines would confer protection from lethal AHSV challenge in the horse is justifiable.

## Introduction

African horse sickness (AHS) is a non-contagious, infectious disease of equids caused by African horse sickness virus (AHSV) [1]. It is transmitted by the bite of certain *Culicoides* biting midge species [2]–[4]. In susceptible populations of horses, mortality rates can exceed 90% [5]. Nine different serotypes of the virus have been identified, based on the specificity of its interactions with neutralising antibodies in serum neutralisation assays [6].

The AHSV genome is composed of ten dsRNA segments, which encode seven structural proteins VP 1–7 and four non-structural proteins NS1, NS2, NS3 and NS3a [7]. AHSV particles are organised as three concentric layers of proteins. The outer capsid consists of two proteins VP2 and VP5. VP2 is the principal serotype specific antigen of AHSV, and the majority of neutralising epitopes are located on VP2 [7]–[9]. The virus core, consists of two major proteins, VP7 which forms the core surface layer, and VP3 which forms the innermost ‘subcore’ shell. The subcore surrounds the 10 segments of the viral genome, and contains three minor proteins VP1, VP4 and VP6 that form the core associated transcriptase complexes [7].

AHSV is endemic in tropical and sub-tropical areas of Africa, south of the Sahara [1], but epizootics of AHSV have also occurred outside Africa, resulting in high mortality rates and severe economic loses, such as those reported in the Middle East in 1959, or in North Africa and Spain during 1969 and 1987 [10], [11]. In the latter outbreaks, an extensive vaccination program and movement control measures led to complete eradication of the disease [12], [13].

Vaccination plays an essential role in the control and prevention of the disease and vaccine development has been one of the main focuses of AHS research. Live polyvalent vaccines for AHSV are commercially available in South Africa, and have been developed by cell-culture attenuation of the virus [14]. However, concerns still exist over their use, particularly in those countries where the disease is not endemic because of potential gene segment reassortment between field and vaccine strains, potential reversion to virulence and inability to distinguish vaccinated from infected animals [1], [14]–[17]. In the past, inactivated vaccines have been shown to induce protective immunity [18], [19], but are not readily available.

For these reasons, research has focused on the development of recombinant subunit and virus-like particle AHSV vaccines using baculovirus expression systems. These recombinant vaccines in conjunction with novel diagnostics allow the differentiation between vaccinated and naturally infected animals and may provide homologous protection against AHSV challenge [20], [21]. However, these types of vaccines have yet to be used for commercial vaccine production.

Another strategy that has been used for AHSV and other viral vaccines is the use of live viral vectors. These have the ability to introduce the recombinant gene product into the MHC class-I pathway of antigen presentation and therefore prime cytotoxic T cells as well as generate humoral immunity [22]–[24]. Most recently, recombinant Venezuelan equine encephalitis virus-derived replicon vectors, individually expressing the VP2 and VP5 genes of AHSV-4, have been developed. However, in initial tests these constructs failed to induce neutralizing antibodies in horses [25].

Poxvirus based vectors have been established as a potent system for the development of candidate recombinant vaccines for many viral diseases [26], [27]. In the case of AHSV, the potential of poxvirus vector vaccination has been demonstrated using a recombinant Vaccinia virus (Western Reserve (WR) strain) expressing AHSV-4 VP2 [28]. However, WR strain derived vaccinia viruses still replicate in mammals and some concerns exist over their safety. For this reason, the use of poxvirus vectors with limited replication capacity, are preferred for vaccine development.

The modified vaccinia Ankara (MVA) strain was derived after more than 570 passages in primary chick embryo fibroblasts [29]. The resulting virus has lost the ability to productively infect mammalian cells [30]. Virus replication is blocked at a late stage of morphogenesis in mammalian cells, leaving expression of late, as well as early, viral genes unimpaired [29]. MVA was shown to be non-pathogenic even for immunodeficient animals and recombinant viruses were found to be able to synthesise high levels of a foreign protein in human cells, demonstrating the potential of MVA as a safe and efficient expression vector [29]. Recent studies have also provided evidence for the safety and immunogenicity of recombinant modified vaccinia Ankara (MVA) in ponies [31], [32]. For these reasons recombinant MVA was chosen as a vector for AHSV antigens.

Due to the lethality of AHSV challenge studies and the number of animals that would be required it was deemed important to carry out this pilot investigation to determine whether it is possible to induce an AHSV-specific immune response in ponies by vaccination with recombinant MVA viruses expressing AHSV proteins. For this we constructed three recombinant MVA viruses expressing three antigens of AHSV-4: VP2, VP7 and NS3, and characterised the antibody responses that were generated. These antigens were chosen for several reasons. Studies using recombinant VP2 vaccines in horses have demonstrated that VP2 induces a neutralising antibody response, which is serotype specific, affording protection against homologous virus challenge [21], [28]. AHSV-9 VP7 has been shown to provide protection in the mouse model against a heterologous challenge with a known lethal dose of AHSV-7 [33]. NS3 was chosen as it is known to stimulate antibody responses in the horse [34], [35] and studies with closely related bluetongue (BTV) have demonstrated that NS3 may also be a CTL target for BTV-immune sheep [36], [37].

The results of this study demonstrate the immunogenicity of recombinant MVA vectored AHSV antigens, in particular MVAVP2. Further work with MVANS3 is needed, however, the use of MVAVP2 and MVA VP7 in a lethal challenge study in the future would be justified.

## Results

### Characterisation of recombinant MVA viruses

**Figure 1:**
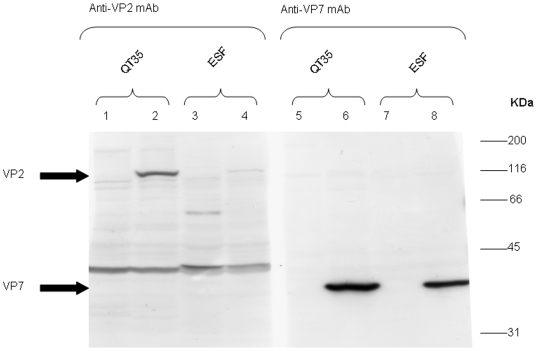
Detection of MVAVP2 or MVAVP7-expressed VP2 and VP7 protein, respectively, within QT35 and ESF cells. Cell lysates of uninfected cells (lanes 1,3,5 and 7) and cells infected at high MOI with MVA-VP2 (lanes 2 and 4) or MVA-VP7 (lanes 6 and 8), and harvested at 24 hours post-infection, were separated by SDS-PAGE on 10% gels. Immunoblotting was conducted with either anti-VP2 mAb (lanes 1–4) or anti-VP7 mAb (lanes 5–8).

**Figure 2:**
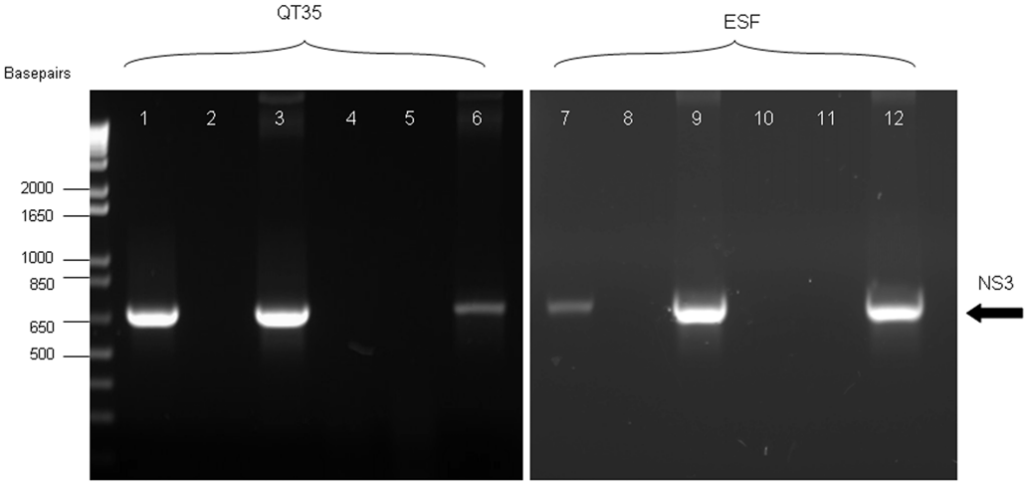
Detection of NS3 RNA transcripts from MVANS3. 1% agarose gels showing RT-PCR and PCR products using RNA templates extracted from MVA infected QT35 and ESF cells 4 hours post infection. Tracks 1–3 QT35 RNA extract RT-PCR products. Tracks 4–6 QT35 RNA extract PCR products. Tracks 7–9 ESF RNA extract RT-PCR products. Tracks 10–12 ESF RNA extract PCR products. Expected product size 670 bp. Tracks 1, 4, 7, & 10 MVANS3; 2, 5, 8, & 11 MVA wild type; 3, 6, 9, & 12 pSC11NS3 plasmid.

Confirmation of gene expression of the recombinant MVA viruses used as vaccines in this study was tested in quail fibrosarcoma (QT35, ECACC Ref. No. 93120832) and equine skin fibroblasts (ESF) infected cells by detection of the expressed AHSV gene product, or, where no antibody was available, detection of specific RNA transcripts. Both VP2 and VP7 were detected in infected cell lysates by western blotting with VP2 and VP7 specific monoclonal antibodies at the expected molecular weights of ∼116 and ∼38 kDa, respectively (Figure 1). The detection signal for VP2 was stronger in QT35 than in ESF cells, revealing a potential difference in expression levels of this protein between these cell lines. Expression of VP7 was also confirmed by indirect immunofluorescence (data not shown). Expression of NS3 protein from MVANS3 was not tested due to a lack of NS3-specific antibody reagents. The functionality of the vaccinia P_7.5_ NS3 expression cassette was confirmed by detection of NS3-specific RNA sequences at 4 hours post-infection in MVANS3 infected QT35 and ESF cells by RT-PCR. The cDNA amplicons generated were the expected size (670 bp). The absence of contaminating genomic DNA in all of the RNA extracts was confirmed by negative PCR results using RNA templates (Figure 2).

### Analysis of antibody responses to MVA

**Figure 3:**
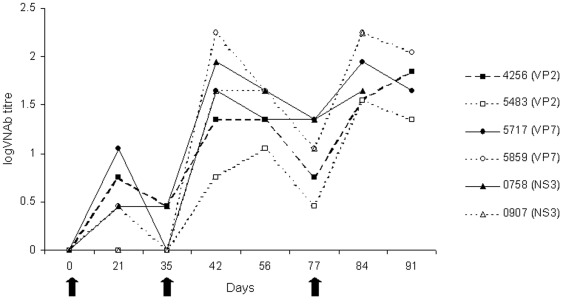
Development of neutralising antibodies against MVA following vaccination. MVA plaque reduction neutralisation titre of sera taken from ponies following initial vaccination with recombinant MVA and two subsequent boosts. Arrows denote days of vaccination.

Following an initial vaccination with recombinant MVA viruses, all of the ponies displayed transient inflammation at the sites of inoculation and slight elevation of rectal temperatures. This was not observed following subsequent inoculations. All the ponies were seronegative for MVA prior to vaccination. Following the initial dose, a slight increase in MVA specific antibody levels was observed by 21 days post vaccination in four animals, but had declined again by day 35. Antibodies against MVA were detected in all animals after the second vaccination (day 35), the serum titres ranging from 0.75 to 2.25 on day 42 but these had declined by day 77. A third dose boosted the antibodies again, and the range of titres was narrower than after the second vaccination (Figure 3).

### Analysis of antibody responses to AHSV antigens

#### Western blots

**Figure 4:**
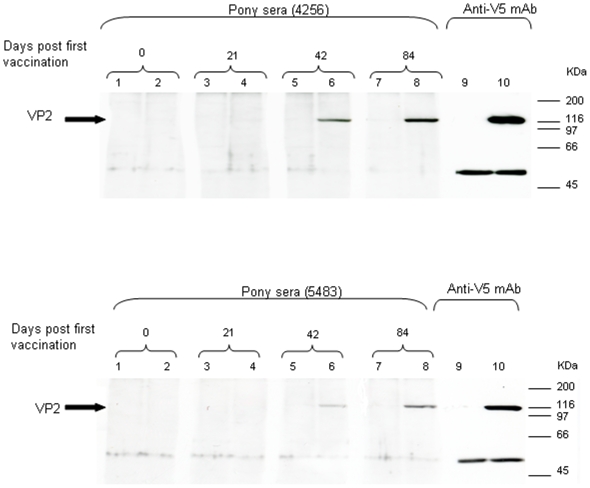
Detection of immunoprecipitated, recombinant-baculovirus-expressed VP2V5 with MVAVP2-vaccinated pony serum. Lysates of uninfected Sf9 cells (lanes 1,3,5,7 and 9) and Sf9 cells infected with recombinant baculovirus FBVP2-V5 were immunoprecipitated with anti-V5tag mAb and Protein G agarose. The immunoprecipitates were separated by SDS-PAGE on 10% gels, and immunoblotted with MVAVP2 vaccinated pony sera (4256, 5483) (lanes 1–8) or anti-V5tag mAb (lanes 9 & 10). The pony sera tested were derived from a pre-vaccination control bleed (lanes 1 & 2) and three post-vaccination bleeds (lanes 3–8) from days 21, 42, and 84, respectively. A non-specific band was present to some extent in each lane at ∼50 KDa.

An antibody response against AHSV VP2 was detected in both MVAVP2 vaccinates by western blotting using sera taken at 42 and 84 days post vaccination (Figure 4). Recombinant baculovirus expressed VP2-V5 was similarly detected at ∼116 kDa with the anti-V5 mAb. For both ponies, the VP2-V5-specific band was more evident using the samples collected on day 84 (after the third vaccination) than on day 42 (after the second vaccination).

**Figure 5:**
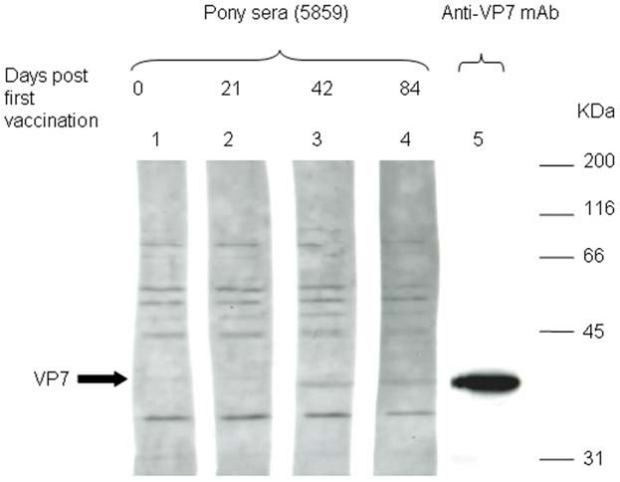
Detection of recombinant baculovirus-expressed VP7 with MVA-VP7-vaccinated pony serum. A semi-purified preparation of recombinant baculovirus FBVP7-expressed VP7 was separated by by SDS-PAGE on 10% gels, and immunoblotted with MVA-VP7-vaccinated pony sera (lanes 1–4) or anti-VP7 mAb (lane 5). The pony sera tested were derived from a pre-vaccination control bleed (lane 1) and three post-vaccination bleeds (lanes 2–4) from days 21, 42, and 84, respectively.

An antibody response against VP7 was detected at 42 and 84 days post vaccination in one of the two MVAVP7 vaccinates (pony 5859) (Figure 5), with a faint band corresponding to VP7 (∼38 kDa) observed for sera collected on days 42 and 84. No antibodies to VP7 were detected in the other MVAVP7 vaccinated pony (5717) (data not shown). No antibodies to immunoprecipitated NS3 were detected in either of the ponies vaccinated with MVANS3 (data not shown).

#### Virus neutralisation test

The virus outer capsid protein VP2 is the dominant AHSV antigen and the main target of homologous virus neutralising (VN) antibodies. Therefore, success of vaccination with MVAVP2 was also assessed by determining whether a VN antibody response was induced. The results of the VN test are shown in Table 1. Both ponies were seronegative prior to vaccination. Following the initial vaccination the serum VN antibody titres in both ponies were low. However, following the first MVAVP2 boost (day 35) VN titres increased rapidly (maximum VN titre of 2). The titres remained at a similar level until at least day 84.

**Table 1:** AHSV-neutralising antibody responses in ponies vaccinated with MVAVP2.

	Post-vaccination titres*	
Day	Pony 5483	Pony 4246
(V1) 0	neg	neg
28	0.4	0.6
(V2) 35	0.3	0.5
42	1.8	1.9
56	1.6	2
(V3) 77	1.6	2
84	1.7	1.7
91	1.35	N.T.
Positive control	2.5	
Negative Control	neg	

* Expressed as the reciprocal of the highest dilution that provided >50% protection of the Vero cell monolayer.

## Discussion

In recent years, there has been an unprecedented emergence of the *Culicoides* transmitted pathogen BTV in Europe and the Mediterranean. This has been attributed to the northward expansion of the major BTV vector, *Culicoides imicola*, possibly influenced by climate change; as well as the involvement of indigenous European *Culicoides* species [38], [39]. Due to the similarities between BT and AHS viruses and their vectors, it has been suggested that should AHSV incur into Europe there is the potential for it to become as widespread as BTV [39], [40]. As there are concerns over the use of modified live AHSV vaccines, the development of efficacious and safer AHSV vaccines, suitable for use in both endemic and non-endemic regions, is therefore an important focus of AHSV research. Poxvirus vectored vaccines, with enhanced safety due to limited replication are of particular interest. Indeed, recombinant canarypox virus based vaccines for the prevention of equine influenza, West Nile virus or equine herpesvirus infections have already been developed for use in horses, and for the prevention of the closely related BTV in sheep [41]–[44]. Vaccination of horses with recombinant MVA based vectors has also been shown to be an effective means of inducing protective immunity to influenza virus infection [32], [45].

A recent study comparing recombinant canarypox and MVA vectors has demonstrated that antigen production by recombinant MVA was greater than that from recombinant canarypox virus in certain mammalian cell lines and primary human cells tested [46]. This observation was primarily due to a longer duration of antigen production in recombinant MVA-infected cells. Antigen production by MVA was also noted to be greater in human dendritic cells, and resulted in enhanced T-cell stimulation in an *in vitro* antigen presentation assay [46]. Therefore, the potential of recombinant MVA viruses, expressing AHSV proteins, to induce AHSV-specific immune response in ponies was investigated.

In this study we successfully generated three recombinant MVA viruses, MVAVP2, MVAVP7 and MVANS3, Western blots using both MVAVP2 and MVAVP7 infected QT35 and ESF cell lysates confirmed that these recombinant viruses were infectious and able to express the AHSV antigens in equine cells. We did not have access to NS3-specific antibodies and therefore it was difficult to evaluate NS3 protein expression in MVA infected cells. However, detection of NS3 specific sequences by RT-PCR from total RNA extracted from QT35 and ESF cells infected with MVANS3 suggested the expression cassette was functional and so the recombinant MVANS3 was used in the vaccination studies.

Following vaccination with recombinant MVA viruses, all the ponies remained healthy, although transient inflammation of the inoculation sites and elevation of rectal temperatures were observed after the initial vaccinations. This is suggestive of an active infection, and was probably associated with the MVA vector, as subsequent inoculations did not induce these reactions indicating pre-existing immunity to the MVA. Indeed, MVA-specific neutralising antibody responses were demonstrated in all ponies after the initial vaccination. After subsequent vaccinations all the ponies showed an anamnestic response and produced higher levels of MVA neutralising antibodies. This is consistent with other work carried out in the horse [31].

Following the second vaccination (day 42), VP2 specific antibody responses were demonstrated for both MVAVP2-vaccinated ponies by western blotting. The neutralising activity of the antibody response was confirmed by AHSV VN test, with titres rising to 1.8/2 units (ponies 5843 & 4246 respectively) after the second vaccination. This is a promising result, because in a previous study using recombinant vaccinia expressing AHSV-4 VP2, significant neutralising titres were not observed in vaccinated horses until after the third inoculation [28]. Furthermore, the VN titres observed in this study are at similar levels to those recorded in the literature, which have been observed to provide protection against lethal AHSV challenge [21], [28].

Antibody responses following MVAVP7 immunisation were also observed, albeit in only one of the two vaccinates. In the mouse model, mice vaccinated with recombinant AHSV VP7 have also shown very variable anti-VP7 antibody responses. However, VP7 vaccination still conferred protection against heterologous challenge with a lethal dose of AHSV and it was suggested that this protection was unlikely to be due to the antibody-mediated immune response alone, but may have been related to cell-mediated responses [33]. Additional work to investigate whether this is the case in the horse is therefore required.

Neither of the ponies vaccinated with MVANS3 developed NS3 specific antibody responses during the study period, despite showing clear neutralising antibody responses to the MVA vector. Although NS3 mRNA could be demonstrated *in vitro*, the expression of the NS3 protein could not be confirmed. It is possible that the protein was expressed at very low levels due to rapid degradation after translation. Previous studies of BTV-NS3 have indicated that it is poorly expressed in mammalian systems infected with BTV although it is expressed well in insect cell systems [47], [48]. The low expression of NS3 in mammalian cells may therefore be a normal property of the AHSV Segment-10 mRNA.

The impact of previous immunity to both replication competent and replication deficient vaccinia virus has been investigated in several studies and is suggested to reduce the effectiveness of subsequent vaccinations [22], [24], [49], [50]. The second MVAVP2 vaccination in this study was found to boost VP2 specific antibody titres, despite a pre-existing antibody response to the MVA vector, although the third vaccination did not increase the VNAb response any further. Therefore, it may be necessary to investigate methods to enhance immune responses generated against the AHSV antigens. The use of DNA priming prior to vaccination with recombinant MVA has been used successfully in several studies [24], [51], [52] and could be applied to future work with the recombinant MVA AHSV vaccines.

In summary, the ability of recombinant MVA viruses encoding AHSV proteins (VP2, VP7, and NS3) to induce AHSV antigen-specific antibody responses was investigated. The confirmation of antibody responses against VP2 and VP7, in particular induction of strong virus neutralising antibody by MVAVP2, warrant the further investigation of MVA vectored AHSV vaccines, to investigate whether they also induce cellular immune responses and ultimately induce protection from both homologous and heterologous AHSV challenge.

## Materials and Methods

### Generation of recombinant MVA viruses expressing VP2, VP7 and NS3

The cDNA copies of genome segments 2, 7 and 10 from the Madrid 1987 strain of AHSV-4 (IAH reference collection number: SPA1987/01) [53] were generated using previously described methods [54] and cloned into plasmid pT7-Blue (Novagen, Madison, USA) using standard molecular biology techniques.

The AHSV genes were subcloned into the vaccinia transfer vector pSC11 downstream of the vaccinia P_7.5_ promoter [55], to generate pSC11VP2, pSC11VP7 and pSC11NS3. Integration of the expression cassettes into the thymidine kinase (TK) locus of MVA, was achieved by infecting QT35 cells grown in Glasgow minimum essential medium (GMEM) supplemented with 10% foetal calf serum, 10% tryptose phosphate broth, 2 mM L-glutamine, 100 units/ml penicillin and 100 µg/ml streptomycin (all from Sigma-Aldrich, Dorset, UK), with wild type MVA (MOI 0.05 for 1 h, 37°C) followed by transfection with pSC11VP2, pSC11VP7 or pSC11NS3 using Lipofectamine 2000 (Invitrogen, Paisley, UK). Cells were incubated for 48–72 hours at 37°C, 5% CO_2_. The cells were scraped from the plastic and pelleted at 1500×g for 10 minutes. The pellet was resuspended in 1 ml serum free GMEM and freeze-thawed three times to lyse the cells and release the cell-associated virus particles.

Recombinant viruses, denoted as MVAVP2, MVAVP7 and MVANS3, were purified by selecting for *Lac Z* marker gene expression and TK negative phenotype, using methods that have been described previously [55]. Pure recombinant MVAVP2, MVAVP7 and MVANS3 virus stocks were propagated in QT35 cells, titrated and used for vaccination of ponies. The MVANS3 virus stock was purified once over a sucrose cushion as described previously [32]. MVAVP2 and MVAVP7 virus stocks were not purified before vaccination.

### Confirmation of AHSV gene expression from recombinant MVA

#### MVANS3 Reverse Transcriptase (RT)-PCR

Total RNA was extracted from ESF and QT35 cells infected with the recombinant MVA using RNeasy mini kit (Qiagen, Crawley, UK) and DNase treated with the Turbo DNA-free kit (Ambion, Huntindon, UK). RT-PCR reactions were set up using Superscript one-step RT-PCR kit (Invitrogen), with 5 µl RNA template, using NS3 specific primers NS3f (5′-ACTGTGGATCCTCATGAATCTAGCTGCAA-3′) and NS3r (5′-GATACGAATTCCTAGCTTTCGCCATAC-3′) as forward and reverse primers, respectively. A PCR was also set up using the same primer set as for the RT-PCR to confirm the absence of DNA in the samples.

### Analysis of VP2 and VP7 expression by recombinant MVA using Western blotting

2×10^6^ QT35 cells or ESF were mock infected or infected with MVAVP2 or MVAVP7 (MOI of 3), and harvested at 24 h post-infection. Cell lysates were prepared using 200 µl of ice-cold solubilisation buffer [20 mM Tris-HCL, 150 mM NaCl, 1% sodium deoxycholate, 1% Tergitol, 0.1%, SDS, 2 mM EDTA (all supplied by Sigma-Aldrich), supplemented with protease inhibitors (Roche, Mannheim, Germany) prior to use], centrifuged at 17900×*g* for 15 minutes at 4°C, and the supernatants retained.

Lysates were mixed 1∶1 with Laemmli sample buffer, heated at 95°C for 5 minutes, and separated by SDS-PAGE on 10% polyacrylamide gels. The separated protein was transferred to nitrocellulose, blocked overnight at 4°C with 0.05% Tween20/phosphate buffered saline (PBS-T) supplemented with 5% bovine serum albumin (Sigma-Aldrich) and 5% non-fat milk powder, prior to immunodetection. Blots were probed with monoclonal antibodies (mAb) 8BC2 and 10AB1 specific for VP2 and VP7 of AHSV-4 respectively [56], [57] provided by INGENASA (Madrid, Spain). Following extensive washing with PBS-T, membranes were incubated with a goat anti-mouse horseradish peroxidase (HRP)-conjugated secondary antibody (Dako, Glostrup, Denmark) diluted 1∶1000 in blocking buffer, again washed thoroughly in PBS-T, and bound antibodies visualised with an enhanced chemiluminescence kit (Amersham Biosciences, Little Chalfont, UK). Protein separation was visualized with rainbow pre-stained markers (Bio-Rad Laboratories, Hemel Hempstead, UK) and biotinylated protein markers (Bio-Rad Laboratories) were additionally used for marker-visualisation on ECL Hyperfilm (Amersham Biosciences).

### Generation of recombinant baculoviruses expressing AHSV proteins

A baculovirus expression system was used in order to obtain a source of antigens for use in immunological assays. The VP2, VP7 and NS3 genes were PCR amplified with gene specific primers containing restriction enzyme recognition sequences. The VP2 was amplified using primers 5′-CTTGAATTCGGACCATGGCGTCCGAGTTTGGAATATTG-3′ and 5′-GAAGAATTCCTTCCGTTTTTGCGAGTAACTTCG-3′; VP7 was amplified with primers 5′-GTTCGCGGCCGCACCATGGACGCGATAGCAGCAAG-3′ and 5′-GTTCGCGGCCGCAATCTAGTGGTAGGCTGCTAG-3′; and NS3 was amplified with 5′-ATCGGATCCACCATGAATCTAGCTG-3′ and 5′-ATATTCTCGAGTGCTTTCGCCATACT-3′. In the case of VP2 and NS3 the genes were antigenically tagged. The carboxyterminal V5 antigen tags were generated by subcloning the PCR amplicons into the MCS site of plasmid pcDNA6/V5-His C (Invitrogen) in frame with the tag sequences. Subsequently the pcDNA6VP2 and pcDNA6NS3 were digested with PmeI (New England Biolabs, Ipswich, UK) and the V5 tagged versions of VP2 and NS3 ligated into the MCS site of pVL1393 (Invitrogen) downstream of the polyhedrin promoter. In the case of VP7, the amplicon was digested directly with restriction enzyme and cloned into the MCS site of pVL1393.

Recombinant baculoviruses were generated by homologous recombination, according to manufacturer's instructions, using flashBAC (NextGen Sciences Ltd, Huntingdon, UK), a baculovirus circular DNA lacking part of the essential gene ORF1629. Briefly, Sf9 cells seeded in EX-CELL 420 serum-free medium (Sigma-Aldrich) were co-transfected with the baculovirus pVL1393 shuttle vectors, Cellfectin (Invitrogen) and flashBAC and incubated for 5 days at 28°C. Following the incubation period the medium containing the seed stock of recombinant baculovirus was harvested.

The recombinant baculoviruses, denoted as FBVP2-V5, FBVP7 and FBNS3-V5 were grown in Sf9 cells, cells pelleted by low speed centrifugation and lysed in solubilisation buffer. This was used as an antigen source in Western blots to analyse the antibody responses of the vaccinated ponies.

### Ponies, vaccination procedures and sampling

Six Welsh mountain ponies were divided into three groups of two animals, each group being vaccinated with MVAVP2 (ponies 4256 & 5483), MVAVP7 (ponies 5859 & 5717) or MVANS3 (ponies 0758 & 0907). Vaccinations were performed by administering a dose of 10^8^ plaque forming units (pfu) on days 0, 35 and 77, using a combination of intramuscular and intradermal routes. Intradermal vaccination was performed using a needle-less injection device (Injex, Anaheim, USA). Serum samples were collected weekly during the vaccination study for analysis of the antibody response. All work involving experimental ponies was performed under a Home Office Project Licence and had been approved by the Animal Health Trust's Ethical Review Committee.

### Analysis of MVA-specific antibody responses by plaque reduction neutralisation test (PRNT)

Anti-vaccinia MVA antibody responses were analysed in the vaccinated animals by a plaque reduction neutralisation test. Doubling dilutions of serum samples collected from the vaccinated animals were made in 100 µl volume of serum-free growth medium in triplicate wells of 96-well plates and incubated with 10^2^ pfu/100 µl of MVANS3 for 2 hours at 37°C, 5% CO2. One hundred microlitres of the sera-virus suspension was then added to 96-well plates containing pre-formed monolayers of QT35 cells. After 2 hours incubation 100 µl of growth medium was added and the plates incubated overnight at 37°C, 5% CO_2_. The following day the medium was removed from the wells, the cells fixed with 0.5% glutaraldehyde (Sigma-Aldrich), washed twice with 2 mM magnesium chloride (Sigma-Aldrich) in PBS and stained with an X-gal sensitive stain (10 mM potassium ferricyanide/ferrocyanide, 0.02% Tergitol, 0.01% sodium deoxycholate, 0.5 mg/ml X-gal, 2 mM magnesium chloride in PBS). After 30 minutes incubation at 37°C, the plates were washed again and the number of blue plaques formed in each well was determined. Virus infectivity was considered to be neutralised in each well when more than a 90% reduction in the number of plaques was observed relative to the positive control. Serum titres were calculated according to the Karber formula [58], the highest serum dilution showing virus neutralisation being considered as the end-point.

### Analysis of AHSV-specific antibody responses by Western blotting

An immunoprecipitation method was employed to obtain a partially purified and concentrated source of VP2 or NS3 antigen, respectively. Uninfected, FBVP2-V5 or FBNS3-V5-infected Sf9 cell lysates were immunoprecipitated with anti-V5 mAb. Lysates were first pre-adsorbed against Protein G agarose (Calbiochem, Darmstadt, Germany) and then mixed overnight at 4°C with Protein G agarose and anti-V5 mAb (Invitrogen), according to the manufacturer's guidelines. Samples were washed four times with solubilisation buffer, then mixed 1∶1 with 2× Laemmli sample buffer. The immunoprecipitates were eluted from the protein G agarose by heating at 85°C for 5 minutes. The antigen source for VP7 consisted of partially purified VP7 crystals, generated using previously described methods [59]. Immunoprecipitates of VP2 and NS3, and purified VP7 were separated by SDS-page on 10% polyacrylamide gels, and Western blotting conducted as described above.

Blots were probed with equine serum samples (1∶100) or mAb to VP2 (undiluted), VP7 (1∶100), or anti-V5 antibody (1∶5000), diluted in blocking buffer. Equine sera labelled membranes were probed with a goat anti-horse HRP-conjugated secondary antibody diluted 1∶10,000 in blocking buffer (Jackson ImmunoResearch Laboratories, West Grove, USA).

### Analysis of AHSV-VP2-specific antibody responses by AHSV virus neutralisation test

Serum samples from the MVAVP2 vaccinates, AHSV seropositive (positive control) and AHSV seronegative horses (negative control) were heat inactivated for 30 minutes at 56°C, and serially diluted in quadruplicate wells of a 96-well microtitre plates using DMEM [Dulbecco modified minimum essential medium supplemented with 0.6% Penicillin/Streptomycin (all supplied by Sigma-Aldrich)] as diluent. The serum dilutions were incubated with 100 TCID_50_ of AHSV serotype 4 at 37°C for 1 hour and then overnight at 4°C. A virus titration in the absence of horse serum was made to confirm that the virus dose per well was 100 TCID_50_. The following day 2×10^4^ Vero cells in RPMI medium supplemented with 0.6% Penicillin/Streptomycin, 0.6% L-glutamine and 5% adult bovine serum (all supplied by Sigma-Aldrich), were added to every well and the plates incubated for 3 days at 37°C, 5% CO_2_. The plates were checked for AHSV cytopathic effect (cpe), with the end-point of the assay being taken as the highest dilution that prevented AHSV cpe in the wells. Serum titres were then calculated by the Karber formula [58].
